# Neural and cardiac injury markers in fetal growth restriction and their relation to perinatal outcomes

**DOI:** 10.4274/tjod.galenos.2019.84665

**Published:** 2019-03-27

**Authors:** Betül Yakıştıran, Doruk Cevdi Katlan, Tuncay Yüce, Acar Koç

**Affiliations:** 1 University of Health Sciences, Ankara Zekai Tahir Burak Woman’s Health Training and Research Hospital, Clinic of Obstetrics and Gynecology, Ankara, Turkey; 2 İstanbul Süleymaniye Women’s Health Training and Research Hospital, Clinic of Obstetrics and Gynecology, İstanbul, Turkey; 3 Ankara University Faculty of Medicine, Department of Obstetrics and Gynecology, Ankara, Turkey

**Keywords:** Fetal growth disorders, NSE, Troponin T, pregnancy outcome

## Abstract

**Objective:**

To compare the levels of umbilical cord blood Neuron-Specific Enolase (NSE) and troponin T and venous blood gas samples between healthy newborns and growth-retarded fetuses with impaired Doppler velocity or low APGAR scores.

**Materials and Methods:**

This study was a prospective cohort study. The study group comprised 26 patients with intrauterine growth restriction and pathologic Doppler symptoms, and the control group included 24 healthy fetuses. Umbilical cord blood and blood gas samples were taken from all patients. The blood samples were centrifuged and sent to a laboratory to study NSE and troponin T Perinatal outcomes were evaluated from the medical records of the newborns.

**Results:**

Both groups were similar in terms of demographic characteristics. Fetuses with fetal growth restriction (FGR) were born earlier and had lower APGAR scores than the study group. Chronic hypoxemic fetuses in the study group had lower cord pH and HCO_3_ levels. Further, troponin T levels were higher in the study group than in the control group. There were no major differences in Doppler velocity measurements.

**Conclusion:**

It has been understood that cardiac and neuronal injury detection on fetuses with FGR, troponin T, and NSE are indicators that can be used. In the literature there are studies with heterogeneous paradigms using different indicators to find neuronal injury. As a result of this study, it is clear that to assess neonatal prognosis, wider-scoped and comparative studies will provide more information about the subject.

**PRECIS:** NSE and troponin T in fetal growth restriction.

## Introduction

Fetal growth restriction (FGR) is defined as a fetus’s failure to achieve its previously planned growth potential. In most cases, it is secondary to placental insufficiency, and these cases refer to late-onset growth restriction^(1)^. Although the common belief is that placental insufficiency causes FGR, it may be a result of different, unknown etiologic factors.

Fetuses with growth restriction respond to inadequate nutrient and oxygen uptake with abnormal functioning of the endocrine, cardiovascular, hematologic, and neuronal systems. The fetus can experience numerous complications in the neonatal period, including mortality, necrotizing enterocolitis, neonatal asphyxia, meconium aspiration, hypoglycemia, and other metabolic abnormalities^(2)^. Among these complications, impaired cognitive function is the most important because of its hazardous effect on the neonate’s life. Furthermore, altered cardiovascular function in fetal life is a significant risk factor for chronic hypertension and ischemic heart disease for the subsequent life^(3)^. Therefore, to predict and prevent these complications, neurologic and cardiac biomarkers may be helpful for management.

In FGR, the transplacental nutrient flow is diverted through the ductus venosus, initially reaching the brain and heart; this is a brain-sparing effect to protect the blood supply to the vital organs. A fall-off in the interval growth of the abdominal circumference because of altered blood distribution is one of the earliest finding in FGR. Moreover, consumption of different energy substrates is associated with decreased myelination, neurotransmitters, and new synapses^(4)^. Intrauterine hypoxia is the main reason for neural damage in cases of FGR. When neural damage occurs, enzymes are released by the injured neurons and other neuronal system cells, such as astrocytes and Schwann cells. The enzymes are released by villous and intermediate trophoblasts. The most studied markers are neuron-specific enolase (NSE) and S-100B protein^(5)^.

In addition to the neural injury, in growth-retarded fetuses with increased placental resistance, the lengths of the aortic and pulmonary systolic and pulmonary peak velocities shorten, but the length of the aortic peak velocity extends. The ejection fractions of both ventricles are decreased, and this is associated with poor neonatal outcomes and fetal acidosis. Myocardial hypertrophy without ventricular dilatation results from the increased ratio of fetal heart weight to body weight. The cardiac-sparing effect includes all these changes^(6)^ and cardiac ischemia and myocardial necrosis may occur in asphyxiated fetuses^(7)^. Increased levels of troponin T, a cardiac enzyme, are related to cardiac injury. The aim of this study was to compare the levels of umbilical cord blood NSE and troponin T and venous blood gas samples between healthy newborns and growth-retarded fetuses with impaired Doppler velocity or low APGAR scores.

## Materials and Methods

This prospective cohort study was approved by the Clinical Research Ethics Committee of Ankara University (Number of IRB: 05-216-15/23.03.2015). Informed consent was provided by all participants prior to the baseline interview in the original study.

### Patients

We investigated 50 fetuses, 26 of which had been diagnosed as havig growth retardation in the Department of Obstetrics and Gynecology, Ankara University School of Medicine, from 2014 to 2016. The pregnant mothers attended routine antenatal obstetric care throughout their pregnancies and delivered in the same hospital. In the study group, 26 fetuses were diagnosed as having FGR by a perinatologist. The control group included 24 healthy fetuses with no signs of intrauterine hypoxemia or growth abnormalities, as shown by ultrasonography.

### Inclusion and exclusion criteria

The study group’s inclusion criteria were as follows: 1) women who had agreed to participate in the study and had given informed consent, 2) fetuses with signs of chronic hypoxemia and pathologic arterial/venous Doppler velocity measurements and that were born with low APGAR scores, 3) oligohydramnios, 4) gestational age more than 24 weeks, and 5) delivery by cesarean section. The exclusion criteria for both groups were as follows: 1) fetuses with congenital abnormalities and aneuploidy, 2) vaginal/operative births, and 3) preterm rupture of membranes.

### Study parameters

In this study, the patients’ characteristics, and medical and obstetric histories were recorded. In the third trimester of pregnancy, biometric measurements and biophysical profiles were assessed using high-resolution grayscale ultrasonography, and umbilical artery ductus venosus and middle cerebral artery Doppler velocity indices were measured with pulsed-waved and color Doppler sonography. The decision concerning delivery time was made on impaired biophysical profiles and Doppler velocity. Cesarean section was selected as the delivery mode for avoiding the influence of vaginal birth on the blood parameters. Immediately after the baby was delivered by cesarean section, the umbilical cord was clamped and blood gas samples were taken using a heparinized injector. The blood gas samples from the umbilical cord vein were collected and sent for analyses. At the same time, approximately 10 mL of umbilical blood was taken into two empty biochemistry tubes (Isotherm, clot activator/6 mL; Hongyu Med Devices Co Ltd.; Weihai, China), and after 30 minutes, the umbilical blood samples were centrifuged at 3500 rpm for 5 minutes, and the serums were frozen at -80 ℃ in a deep freezer to be kept at -20 ℃. The samples were sent to Düzen Laboratory to study the NSE and troponin T, and they were investigated using electroluminescence immunoassays. The pO_2_, pCO_2_, HCO_3_, and base excess values were studied; gestational age at delivery, birth weight, APGAR 1/5 scores and necessity for neonatal intensive care unit stays were also recorded.

### Statistical Analysis

Data were calculated using the SPSS 11.5 software package for Windows (SPSS Inc., USA). A value of p<.05 was considered to indicate statistical significance. Patients from both groups were compared using the Mann-Whitney U/Student *t-*test, depending on the character of distribution. Categorical variables were compared using the chi-square test and Pearson/Spearman analyses. Linear regression was used to identify the correlation between dependent variables (NSE and troponin T) and independent quantitative parameters.

## Results

A total of 50 fetuses were included in this analysis. Among the pregnancies, for the study group, 26 (52%) fetuses were followed with a diagnosis of FGR or pathologic Doppler symptoms, and for the control group, 24 (48%) healthy fetuses were included. The maternal characteristics, gestational age at delivery, birth weight, and APGAR 1/5 scores are shown in Table 1. Both groups were similar in terms of maternal age and numbers of gravida, livebirths, and abortus. Fetuses with FGR were born earlier and had lower APGAR scores than the study group.

Umbilical cord gas analyses, Doppler velocity indices, and NSE and troponin T levels are summarized in Table 2. Chronic hypoxemic fetuses in the study group had lower cord pH and HCO_3_ levels. Further, troponin T levels were higher in the study group than in the control group. There were no major differences in Doppler velocity measurements.

There was no correlation between NSE and birth-weight, APGAR scores, cord gas analysis, UA, DV, and melting curve analysis Doppler PI. Further, there was a statistically significant difference between troponin T and APGAR scores, pH, HCO_3_, and UA-PI (Table 3).

NSE, hemolysis index, and the DV-PI correlation are summarized in Table 4. NSE and hemolysis index had a positive correlation.

## Discussion

Findings from our study indicated that the FGR group had higher troponin T levels and lower values of pH, HCO_3_,  APGAR scores, birth weight, and gestational age at birth than in the healthy control group. No significant difference was found for NSE levels between both groups.

It is known that placental insufficiency causes altered fetal cardiac output distribution^(8)^. This alteration is with increased placental resistance and cardiac output volume from the right ventricle, and decreased systemic vasoconstriction and normal/elevated left ventricle cardiac output^(6,8)^. In healthy pregnancies, right ventricle output increases with gestational age. This physiologic alteration for growth-restricted fetuses causes a decrease in ejection of the right ventricle, increase of systemic venous pressured pulsatility of the inferior vena cava, hepatic veins, and atrial contraction of the ductus venosus^(9)^. In our results, increased troponin T levels in the study group had a relation between myocardial injury that developed secondary to cardiac alterations and possible cardiac dysfunction. Makikallio et al.^(10) ^reported elevated troponin T levels in fetuses who had umbilical vein atrial pulsatility. Nomura et al.^(11)^ reported a significant relation between troponin T levels and increased ductus venous PIV in Doppler measurements.

Acidemy (pH<7) and elevated base-excess values in cord blood gas analysis is a predictive laboratory test to evaluate neonatal encephalopathy for acute intrapartum hypoxia^(12)^. Fetal acidemia causes neuronal necrosis, apoptosis, and impaired cognitive functions that affects the whole life of newborn. Sheikh and Cantu’s study found that venous pH, base-excess were more predictive than other blood gas parameters for fetal acidemia^(12,13)^. Our results are similar to those of previous studies. Similarly, lower umbilical cord venous pH values and increased HCO_3_ levels were related with increased troponin T levels. However, Yıldırım et al^(7)^. reported that HCO_3_ had no effect on troponin T, but gestational age at delivery and pCO_2_ were associated with increased troponin T levels. In the literature, elevated troponin T levels have been studied on pregnancies treated with long-term tocolytics and fetuses with respiratory distress syndrome, but it has been investigated in a limited number of studies to predict cardiac injury. Creatine kinase, troponin I and T have been studied to evaluate cardiac dysfunction in hypoxemic-ischemic encephalopathy. Yıldırım et al^(7)^. reported that there was a correlation between troponin T and creatine kinase-MB (CKMB), but troponin T was more specific than CKMB.

Although our study supports the existing data linking troponin T and gestational age at delivery, birth weight, APGAR scores, and umbilical artery Doppler PI, Karadeniz et al^(14)^. certified no relationship between troponin T and gestational age or birth weight in fetuses with mild pre-eclampsia. As they mentioned in their report, this could be considered there was insufficient time for elevation of the troponin T plasma concentration.

Perinatal asphyxia and hypoxic ischemic encephalopathy are associated with increased morbidity and mortality rates. As discussed above, several biomarkers (S100, CK-BB, GFAP, NFp) have been investigated to identify neuronal damage in FGR^(5,15,16,17,18)^. However, even today, the most specific and optimal biomarker remains controversial. We observed higher values of NSE in fetuses complicated by FGR, but there was no statistically significant difference. Velipaşaoğlu et al^(19)^. published a cohort study with an association between neuronal injury markers and intrauterine growth restriction; they found no significance for NSE, but they observed a positive correlation for umbilical artery PI, RI, ductus venosus RI, S/d ratio with NSE. We found an association between ductus venosus PI, the hemolysis index, and NSE; also, higher NSE levels were associated with lower APGAR scores. Çeltik et al^(20)^. found that NSE concentrations ascended six hours after delivery for newborns complicated by grade 3 hypoxic ischemic encephalopathies. This results can explain why NSE levels did not increase significantly in our study. It is a fact that premature and small fetuses have lower blood volume and flow than term fetuses. Also, using an injector to take blood samples can result with hemolysis and elevated NSE levels from erythrocytes. The association between NSE and the hemolysis index can explain this. To exclude this determinant, Zinsmeyer et al^(21)^. studied amniotic fluid and reported that amniotic NSE increased in acute hypoxemia. Berger and Richichi^(22)^ concentrated on a formula to exclude the hemolysis index and clinical use of NSE for hemolyzed serum. Our study group contained only a small number fetuses so we did not use this formula.

Some disadvantages exist in our study. First, only small number of fetuses met all criteria for inclusion in the study, and second, only two biomarkers were studied to investigate cardiac and neuronal injury. However previous studies concentrated on the progression of these markers after delivery in newborns, a limited number of studies used these markers on fetuses.

In light of our findings, it has been understood that troponin T and NSE can be used as indicators for cardiac and neuronal injury detection in fetuses with FGR. According to the literature, it can be seen that there are studies with heterogeneous paradigms using different indicators to find neuronal injury. As a result of this study, it is clear that to assess neonatal prognosis, wider-scoped and comparative studies will provide more information about the subject.

## Figures and Tables

**Table 1:**
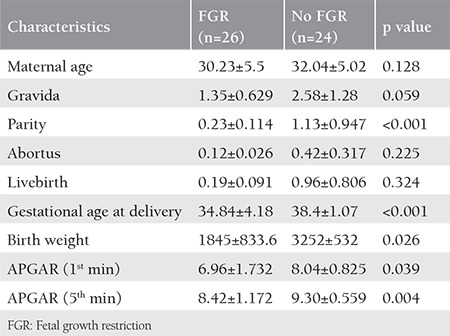
Maternal characteristics and parameters of birth among women with and without FGR

**Table 2:**
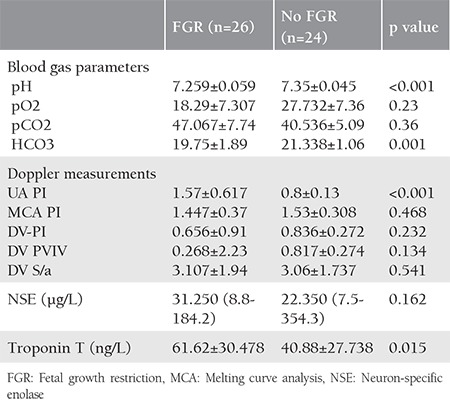
Blood gas analyses and Doppler velocity measurements in groups with and without FGR

**Table 3:**
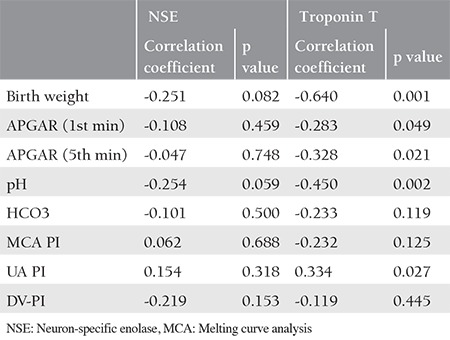
Correlation of NSE and troponin T between birth parameters

**Table 4:**
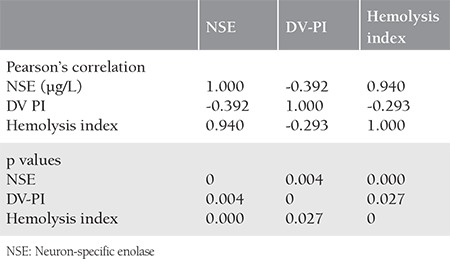
Correlation of NSE, DV, PI, and hemolysis index
